# Genomic landscape of diploid and aneuploid microsatellite stable early onset colorectal cancer

**DOI:** 10.1038/s41598-024-59398-5

**Published:** 2024-04-23

**Authors:** Yumei Zhou, Xianfeng Chen, Jun Chen, Conner D. Kendrick, Ramesh K. Ramanathan, Rondell P. Graham, Kimberlee F. Kossick, Lisa A. Boardman, Michael T. Barrett

**Affiliations:** 1 Department of Research, Mayo Clinic in Arizona, Scottsdale, AZ USA; 2 Division of Computational Biology, Department of Quantitative Health Sciences, Mayo Clinic, Rochester, MN 55905 USA; 3 Division of Gastroenterology and Hepatology, Mayo Clinic, Rochester, MN 55905 USA; 4 Mayo Clinic Cancer Center, Phoenix, AZ 85054 USA; 5 Anatomic Pathology, Mayo Clinic, Rochester, MN 55905 USA; 6 Department of Molecular Pharmacology and Experimental Therapeutics, Mayo Clinic in Arizona, Scottsdale, AZ USA; 7 Present Address: Ironwood Cancer and Research Center, Scottsdale, AZ 85260 USA

**Keywords:** Early onset colorectal cancer, Mutational signatures, Aneuploid tumors, Diploid tumors, Cancer, Oncology

## Abstract

Although colorectal cancer (CRC) remains the second leading cause of cancer-related death in the United States, the overall incidence and mortality from the disease have declined in recent decades. In contrast, there has been a steady increase in the incidence of CRC in individuals under 50 years of age. Hereditary syndromes contribute disproportionately to early onset CRC (EOCRC). These include microsatellite instability high (MSI+) tumors arising in patients with Lynch Syndrome. However, most EOCRCs are not associated with familial syndromes or MSI+ genotypes. Comprehensive genomic profiling has provided the basis of improved more personalized treatments for older CRC patients. However, less is known about the basis of sporadic EOCRC. To define the genomic landscape of EOCRC we used DNA content flow sorting to isolate diploid and aneuploid tumor fractions from 21 non-hereditary cases. We then generated whole exome mutational profiles for each case and whole genome copy number, telomere length, and EGFR immunohistochemistry (IHC) analyses on subsets of samples. These results discriminate the molecular features of diploid and aneuploid EOCRC and provide a basis for larger population-based studies and the development of effective strategies to monitor and treat this emerging disease.

## Introduction

It is estimated that in 2023 there will be 153,020 cases of CRC and 52,550 related deaths in the USA^1^. Notably, the overall incidence and mortality from CRC have declined at a steady rate in recent decades. This reflects the impacts of effective screening programs and more personalized therapies for patients with disease. However, in the last few years any grounds for complacency about CRC disease burden have been severely challenged by reports of highly disturbing increases in CRC incidence in individuals under the age of 50 years^2^. Young age of CRC onset is a hallmark of hereditary CRC syndromes that contribute disproportionately to EOCRCs. The latter include microsatellite instability positive (MSI+) tumors arising in lynch syndrome patients with hereditary non polyposis colorectal cancer (HNPCC). However, the majority of EOCRCs are not associated with familial syndromes or an MSI+ genotype^3^.

CRCs arise in either the left or right side of the colon. The anatomic origin is associated with distinct clinical behavior and molecular features^4^. Studies have reported an enrichment of left-sided tumors arising in younger patients^5^. In older patients, these typically have increased levels of chromosome instability and present with morphology features that are easier to detect in routine screening^4^. However, EOCRCs are diagnosed at more advanced stages of disease and present with distinct histopathological features including mucinous adenocarcinoma, features associated with a worse prognosis^6–8^. Comprehensive genomic profiling has described distinct subsets of late onset CRC^9–12^. Notably, mutational and copy number variant (CNV) profiles from the cancer genome atlas (TCGA) and related studies have identified genomic lesions that “drive” disease. These data confirm that CRCs are a heterogeneous group of tumors that can be subdivided by their molecular features and treated differently, e.g., MSI+ versus microsatellite stable (MSS), *RAS* mutated versus wildtype, *BRAF* mutated versus wildtype, and phosphatidylinositol4,5-bisphosphate 3-kinase (PI3K) mutated versus wildtype. This has provided the basis of improved screening and more personalized treatments for older CRC patients^13,14^. However, to date relatively less is known about the basis of sporadic MSS EOCRCs.

Previous studies of non-inherited EOCRCs have described an enrichment of pathogenic variants in *TP53* and *CTNNB1* and losses of heterozygosity (LOH) at chromosomes 17p and 18q, with relatively low prevalences of mutations in *KRAS*, *BRAF*, and in additional tumor suppressor genes and oncogenes that occur in CRC of older patients^7,15,16^. In addition, these tumors are more often diploid in their DNA content (46% EOCRC vs 26% in later onset CRC), lack widespread chromosome instability, and have a lower frequency of the CpG island methylator phenotype relative to CRCs in older patients^17^. These and other related genetic differences may account for the lack of benefit for adjuvant chemotherapy to date in young adults with CRC in comparison with older adults, and it is likely to be an increasing problem with molecularly targeted agents^18,19^. In contrast, targeted gene panel sequencing of a relatively large cohort of CRC patients did not detect any significant genomic differences between late and early onset CRC^20^.

In order to explore the genomic landscape of EOCRC we applied DNA content based flow cytometry to 21 clinical samples^21,22^. These included a fresh frozen sample from a surgical resection and 20 archived formalin fixed paraffin embedded (FFPE) resected samples from a Mayo Clinic tissue bank. We profiled the exomes of sorted tumor and normal pairs for all 21 cases, the CNVs for a subset of 6 samples, and telomere length in diploid and aneuploid nuclei from 9 cases. Additionally, we screened the 20 FFPE cases for EGFR expression with an established IHC assay. Of significant interest was the comparison of somatic genomic landscapes in diploid and aneuploid tumors and the presence of distinct mutational signatures arising in EOCRC that target signaling pathways of interest and reflect mutational processes operative during their natural history. These results describe shared and unique features of diploid and aneuploid EOCRCs and provide the basis for larger population based investigations of this emerging disease.

## Methods

### Tumor samples

All patients gave written informed consent for collection and use of the samples. The experimental protocol for this study was approved by the Mayo Clinic Institutional Review Board (IRB). All tissue was collected for this study under Mayo Clinic IRB 21-000277, 622-00 and 16-001246. Results from this study were not returned to any of the patients. The use of deidentified archived samples in this study was deemed as minimal risk. All tumor samples were histopathologically evaluated by a board certified GI pathologist prior to genomic analysis (Table 1). All research conformed to the Helsinki Declaration (http://www.wma.net/en/30publications/10policies/b3/).

**Table 1:** Clinical features of EOCRC cohort.

Sample#	Sex	Age at Dx	Pathology	Stage	Site*
EOCRC1	Female	43	ADCA	3	Cecum
EOCRC2	Male	49	ADCA	3	Descending
EOCRC3	Female	46	ADCA	2	Cecum
EOCRC4	Male	39	ADCA	3	Rectosigmoid
EOCRC5	Male	43	ADCA	3	Rectum
EOCRC6	Male	44	ADCA	3	Rectosigmoid
EOCRC7	Female	40	ADCA	2	Cecum
EOCRC8	Male	48	ADCA	2	Rectosigmoid
EOCRC9	Female	41	ADCA	2	Rectum
EOCRC10	Female	44	ADCA	2	Transverse
EOCRC11	Male	31	ADCA	3	Rectum
EOCRC12	Female	49	ADCA	3	Ileocecal valve
EOCRC13	Male	38	ADCA	2	Rectum
EOCRC14	Female	42	ADCA	3	Rectosigmoid
EOCRC15	Male	42	ADCA	2	Cecum
EOCRC16	Male	41	ADCA	3	Cecum
EOCRC17	Female	37	ADCA	3	Sigmoid
EOCRC18	Male	45	ADCA	2	Ascending
EOCRC19	Male	23	ADCA	2	Cecum
EOCRC20	Male	32	ADCA	2	Hepatic flexure
EOCRC21	Male	36	ADCA	2	Sigmoid

*Left side (rectosigmoid, sigmoid, descending, rectum): n = 11, right side (cecum, ileocecal valve, ascending, hepatic flexure): n = 10, transverse: n = 1.

### Flow cytometry

Excess paraffin was removed from each FFPE sample with a scalpel from either side of scrolls then processed according to our published methods^21^. We used one to three 50 µm scroll(s) from each FFPE tissue block to obtain sufficient numbers of intact nuclei for sorting and molecular assays. Frozen tissue samples were minced in the presence of NST buffer and DAPI. Nuclei from each sample, FFPE or frozen tissue, were disaggregated then filtered through a 40 μm mesh prior to flow sorting with an Influx or Aria III cytometer (Becton–Dickinson, San Jose, CA) with ultraviolet excitation and DAPI emission collected at > 450 nm.

### Quality control (Q.C.) measures of single nuclei and of genomic DNA

Nuclei from pre and post sorted samples were inspected with a Countess 3 FL Automated Cell Counter to confirm the quality and yields of each tissue and sorted fraction. DNAs from sorted samples were extracted using Qiagen micro kits (Qiagen Valencia, CA) then assayed with an Agilent Tape Station, to measure the yield and molecular weight of extracted DNA.

### NGS exome

Sorted tumor populations and patient matched control samples were sequenced within the Mayo Clinic Medical Genome Facility (MGF) using established protocols for whole exome analysis^22^. Pair-ended Illumina FASTQ reads were processed with GENOMEGPS—the internal Mayo Clinic secondary data processing pipeline. Briefly, reads QC and adapter trimming were performed by CUTADAPT (https://cutadapt.readthedocs.io/en/stable/), with alignment to reference HG38 by BWA-MEM, followed by reads de-duplication and base quality recalibration by GATK 3.6. For somatic mutation calling from tumor-normal matching samples, MuTect2 from GATK 4.3 were used. The Genome Aggregation Database (gnomAD) was used to build a mutation calling statistics model. For mutation filtering, a panel of normal sequences (somatic-hg38_1000g_pon.hg38.vcf.gz) was used to filter out commonly seen sequencing noise. Orientation biases (i.e., FFPE artefacts) were annotated by a mixture model (LearnReadOrientationModel) from GATK. We used GATK recommended tool (FilterMutectCalls) to filter raw somatic mutations and keep mutations annotated as “PASS” only. We also applied additional filters to reduce false positives including variant allele frequency (VAF) ≥ 10% in tumors, read counts of ≥ 6 for each variant. VCF files were then converted to MAF format with vcftools (https://vcftools.sourceforge.net/) and subjected to R package maftools for tertiary analysis. For mutation signature analysis, we used the default parameters in maftools. The optimal number of signatures was determined using Cophenetic correlation. Extracted signatures were compared to known signatures from COSMIC database, and cosine similarity was calculated to identify best matches in our data.

### Immunohistochemical staining EGFR

Tissue sectioning at 5 μm and IHC staining for the 20 FFPE cases was performed on-line at the pathology research core (Mayo Clinic, Rochester, MN) using the Leica Bond RX stainer (Leica Biosystems). Slides were retrieved for 20 min using BOND Epitope Retrieval Solution 1 (Leica Biosystems). The EGFR primary antibody (Rabbit Monoclonal, Cell Signaling #4267, clone Erb B/Her) was diluted to 1:50 in background reducing Diluent (Dako Products, Agilent) and incubated for 30 min. Slides were incubated for 10 min in DAB and DAB buffer (1:19 mixture) from the bond polymer refine detection system (Leica Biosystems), then rinsed between steps with 1X Bond Wash Buffer. Slides were counterstained for five minutes using Schmidt hematoxylin and molecular biology grade water (1:1 mixture), followed by several rinses in 1X Bond wash buffer and distilled water, rinsed in tap water for three minutes, then dehydrated in increasing concentrations of ethyl alcohol and cleared in 3 changes of xylene prior to permanent coverslipping in xylene-based medium. EGFR scoring was performed by a GI pathologist (RPG) with the following criteria: 0 = no staining, 1+ faint cytoplasmic staining in > 10% tumor cells, 2 + moderate membranous staining, 3 + strong membranous staining. Negative samples included 0 and 1+ cases, while positive samples were 2+ and 3+.

### aCGH

All aCGH was done according to our published protocols^21–24^. Briefly, DNAs from frozen tissue and FFPE samples were treated with DNAse 1 prior to Klenow-based labeling. High molecular weight templates were digested for 30 min while DNAs from FFPE samples were digested for 1 min. In each case 1 µl of 10 × DNase 1 reaction buffer and 2 μl of DNase 1 dilution buffer were added to 7 μl of DNA sample and incubated at room temperature then transferred to 70 °C for 30 min to deactivate DNase 1. Sample and reference templates were then labeled with Cy-5 dUTP and Cy-3 dUTP respectively using a BioPrime labeling kit (Invitrogen, Carlsbad, CA) according to our published protocols^23^. All labeling reactions were assessed using a Nanodrop assay (Nanodrop, Wilmington, DE) prior to mixing and hybridization to 400 k CGH arrays (Agilent Technologies, Santa Clara, CA) for 40 h in a rotating 65 °C oven. After washing microarrays were scanned using an Agilent 2565C DNA scanner and the images were analyzed with Agilent Feature Extraction version 11.0 using default settings. The aCGH data was assessed with a series of QC metrics then analyzed using an aberration detection algorithm (ADM2)^25^.

### Telomere measure

Telomere length measure of sorted nuclei was performed with a monochrome multiplex quantitative polymerase chain reaction (MMqPCR) assay that has been described previously^26^. The MMqPCR assay uses a telomere repeat primer and single copy gene primer for calculation of the relative ratio of telomere quantity based on cycle number to single copy gene (human beta globin gene, HBB) quantity (T/S ratio). Each sample was run in triplicate, and the final T/S ratio was based on the mean of the three measurements.

## Results

### Flow sorting and ploidy

**Figure 1:**
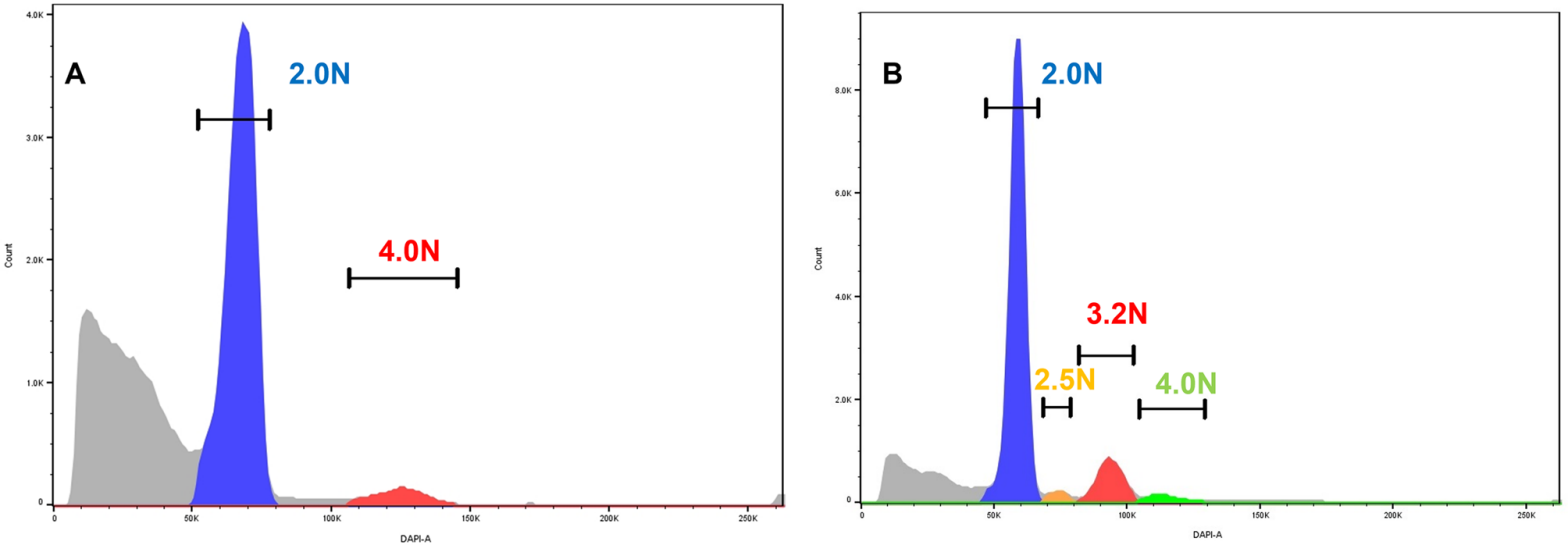
DNA content flow sorting of FFPE EOCRC tissues. (**A**) Diploid tumor EOCRC11 with 2.0N (G_0_/G_1_) peak 2 (P2) and 4.0N (G_2_/M) peak 3 (P3). (**B**) Aneuploid tumor EOCRC12 with diploid (G_0_/G_1_) peak 2 (P2), 4.0N (G_2_/M) peak 5 (P5) and two distinct aneuploid peaks (P3 and P4). Each peak from individual samples was collected during sorting.

We detected aneuploid populations in 11/21 (52%) EOCRC samples. The ploidy of these ranged from near diploid (2.3N) to hyper triploid (3.5N). The remaining 10 tumors were diploid by DNA content flow cytometry. In all cases we collected a minimum of two populations based on their ploidy. These included 2N(G_0_/G_1_) fractions from each tumor, as well as 4N(G_2_/M) and aneuploid fractions when present (Fig. 1). In one case, EOCRC12, we identified and collected a diploid, a 4N(G_2_/M), and two distinct aneuploid populations (2.5N and 3.2N) from the same FFPE sample (Fig. 1B).

### Mutation profiles

**Figure 2:**
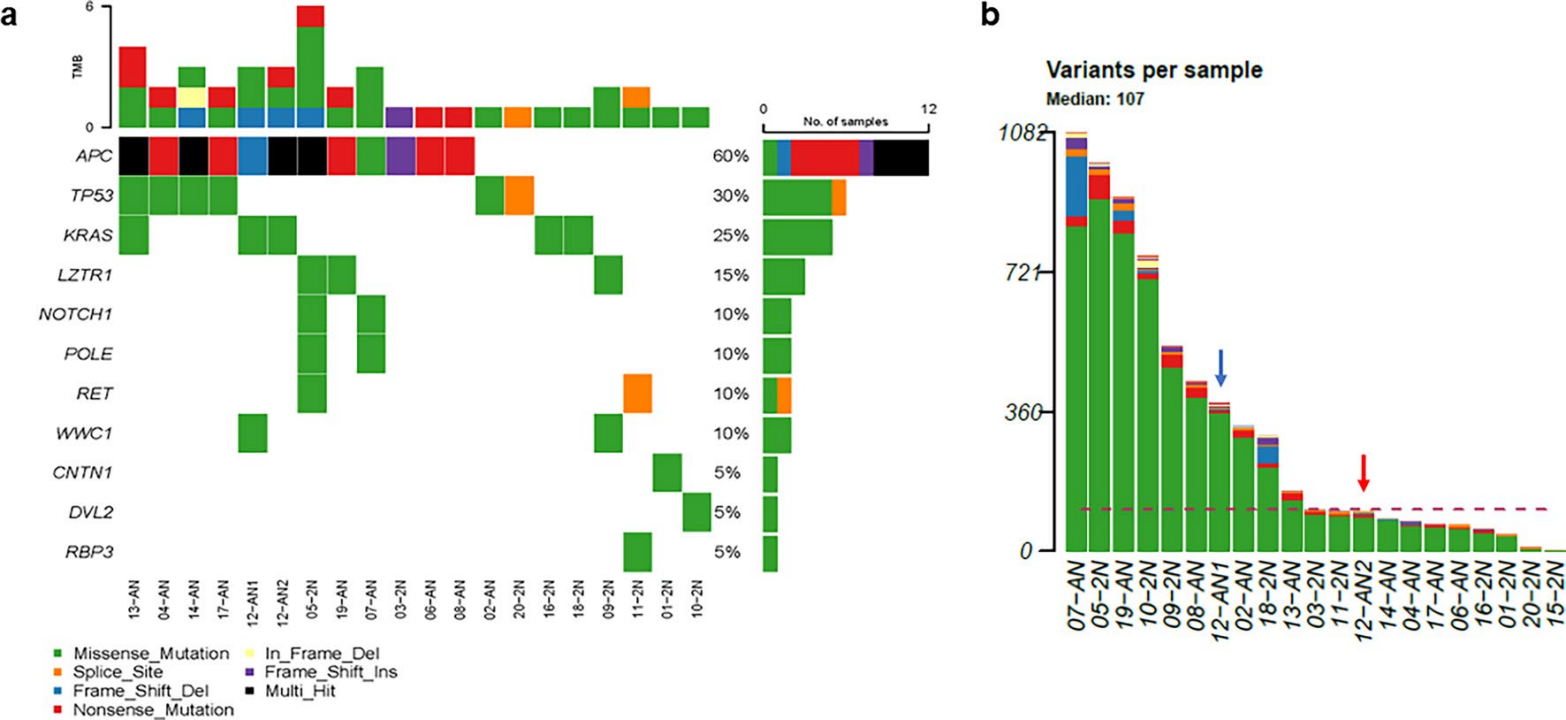
Mutational landscape of EOCRC. (**A**) Oncomap summary of somatic lesions in ASCP genomes. (**B**) Distribution of mutation frequency across EOCRC samples. Blue arrow EOCRC12 AN1, red arrow EOCRC12AN2. Red dashed horizontal line mean number of mutations. Black vertical line.

The exomes of flow sorted populations from the 21 tumor samples were sequenced with our established workflow. These included the two distinct aneuploid populations in EOCRC12. Peripheral blood samples were available from 20 of 21 cases while the sorted diploid fraction was used as patient matched control in EOCRC21. The mean coverage for targeted regions across all 43 normal tumor paired samples was 40 with a range of 12.4–140.1 and a median of 40.5. Pathogenic somatic variants in known driver genes of CRC were detected in diploid and aneuploid tumors (Fig. 2A). These included *APC* (62%), *TP53* (33%), and *KRAS* (24%) (Supplemental Fig. 1A–C). The *APC* variants included 3 cases with R216X and two cases with R283X, both well characterized nonsense variants that are found in familial and sporadic CRCs. The somatic *TP53* variants included missense (n = 5) and nonsense (n = 1) variants targeting the DNA binding domain, as well as a splice site variant, that were present exclusively in aneuploid cases in our cohort. The *KRAS* variants targeted codon 12 in each case including a G12D variant present in both aneuploid populations (AN1, AN2) from EOCRC12. Strikingly, the latter sorted tumor populations from the same biopsy, had distinct TMBs with a > threefold increase in the number of mutations in the 2.5N (AN1) population relative to the 3.2N (AN2) population (Fig. 2B).

In addition, we detected somatic variants in Leucine zipper‐like transcription regulator 1 (*LZTR1*) in three cases (Fig. 2A, Supplemental Fig. 1D). The latter is a Kelch-BTB-BACK domain-containing protein that functions as substrate adaptor of a CRL complex, CRL3LZTR1 implicated in rare neurodevelopmental disorders^27^. Mutations and deletions targeting *LZTR1* have been reported in multiple cancers including colorectal carcinoma^10,12^. The three variants, each a variant of unknown significance (VUS) include G169R that occurs in a highly conserved residue within a KELCH 3 domain, S382L, and L809P adjacent to R810W that compromises LZTR1 protein degradation^28^. Previous studies have shown that pathogenic variants in *LZTR1* fail to promote degradation of EGFR^29^. Thus, the somatic *LZTR1* variants suggested that similar to adult cases, EGFR signalling is activated in EOCRC^30^.

**Figure 3:**
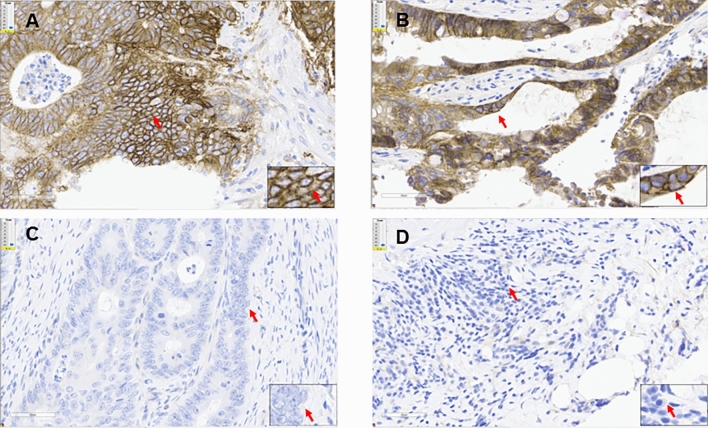
EGFR expression in EOCRC tumors. Immunohistochemistry (IHC) staining for EGFR in tumors with (**A**, **B**) high (3 +) expression (EOCRC19 and EOCRC12), and (**C**, **D**) negative (0) expression (EOCRC5, EOCRC20). Images at 40 × collected with Aperio ImageScope.

EGFR expression was detected by IHC in 14/20 (70%) of cases (Fig. 3, Supplemental Table 2). Based on the IHC labeling-intensity scores, 5 (25%), 4 (20%) and 5 (25%) tumors were scored as 3+, 2+ and 1+, respectively. Notably, 2/3 *LZTR1* mutations, S382L (EOCRC9) and L809P (EOCRC19), were present in samples with 3+ EGFR expression (Fig. 3A-B). In contrast, we did not observe *EGFR* mutations or amplicons suggesting that alternative mechanisms, in addition to *LZTR1* mutations, affect expression. Additional somatic variants included two VUS in *NOTCH1* in EOCRC5 and EOCRC7, and another VUS in the tumor suppressor *WWC1,* a putative regulator of the Hippo/SWH signaling pathway in both EOCRC9 and EOCRC12^31^.

**Figure 4:**
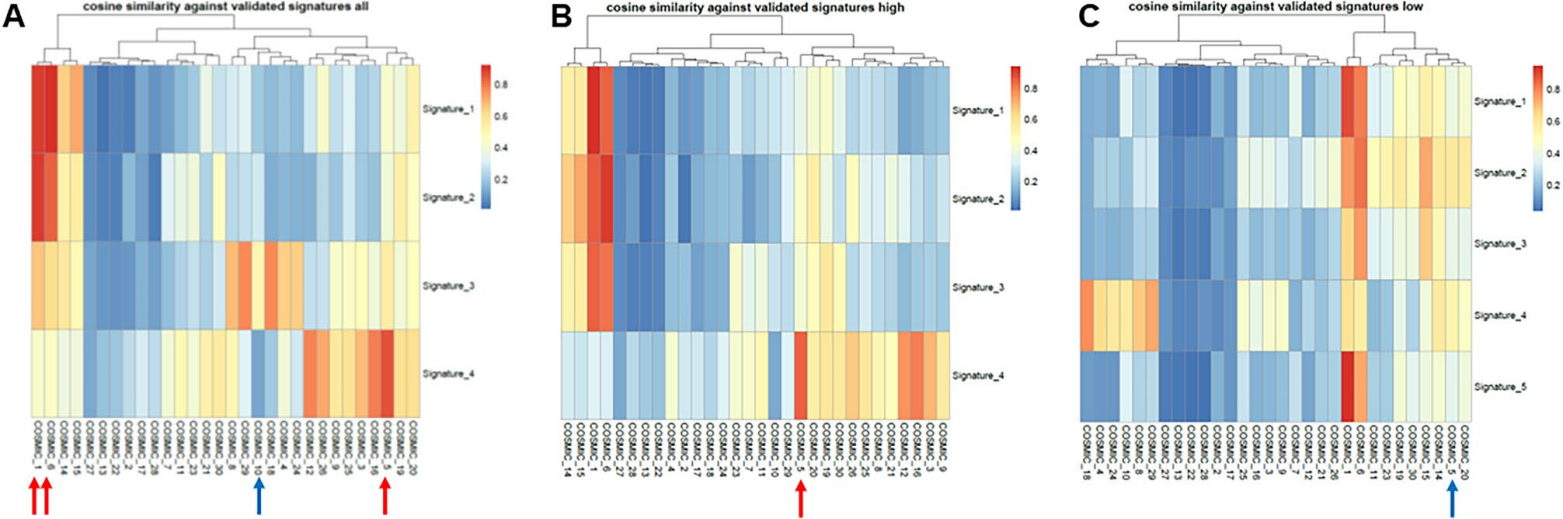
Single base substitution (SBS) mutation signatures of EOCRC. (**A**) SBS 1, 5, and 6 (red arrows) are present, SBS10 (blue arrow) is absent. (**B**) SBS 5 is present (red arrow) in high and (**C**) absent (blue arrow) in low tumor mutation burden (TMB) EOCRC samples.

We detected 3 of the 4 single base substitution (SBS) mutation signatures associated with but not exclusive to CRC in our EOCRC samples (Fig. 4A, Supplemental Fig. 1E)^32^. SBS1 and SBS5 are clock-like signatures that correlate with age whereas SBS6 is one of seven mutational signatures associated with defective DNA mismatch repair and MSI. However, none of the 21 cases were MSI+ based on our exome analyses. Total mutational burden (TMB) distinguished relatively high and low TMB EOCRCs in our cohort that were independent of ploidy and *TP53* mutation (Figs. 2, 4B,C). Notably, SBS5 was present only in higher mutation signature EOCRCs. In contrast, SBS10 with a proposed etiology of polymerase epsilon (*POLE*) exonuclease domain mutations and typically associated with large numbers of somatic mutations (> 100 mutations per Mb) in samples termed hypermutators, was absent in our cohort despite two patients, EOCRC5 and EOCRC7, having somatic variants in *POLE* (Fig. 2). The first of these, R793H, is associated with a germ line polymorphism of uncertain significance (rs1422986795), while the second L1245I, has not been previously reported (Supplemental Fig. 1F). Notably, both of these cases, one diploid tumor and the other an aneuploid tumor, had the highest somatic TMBs including the highest numbers of *POLE* associated variants in their tumor genomes (Fig. 2B)^33^. However, these were below thresholds for SBS10.

### Copy number profiles

**Figure 5:**
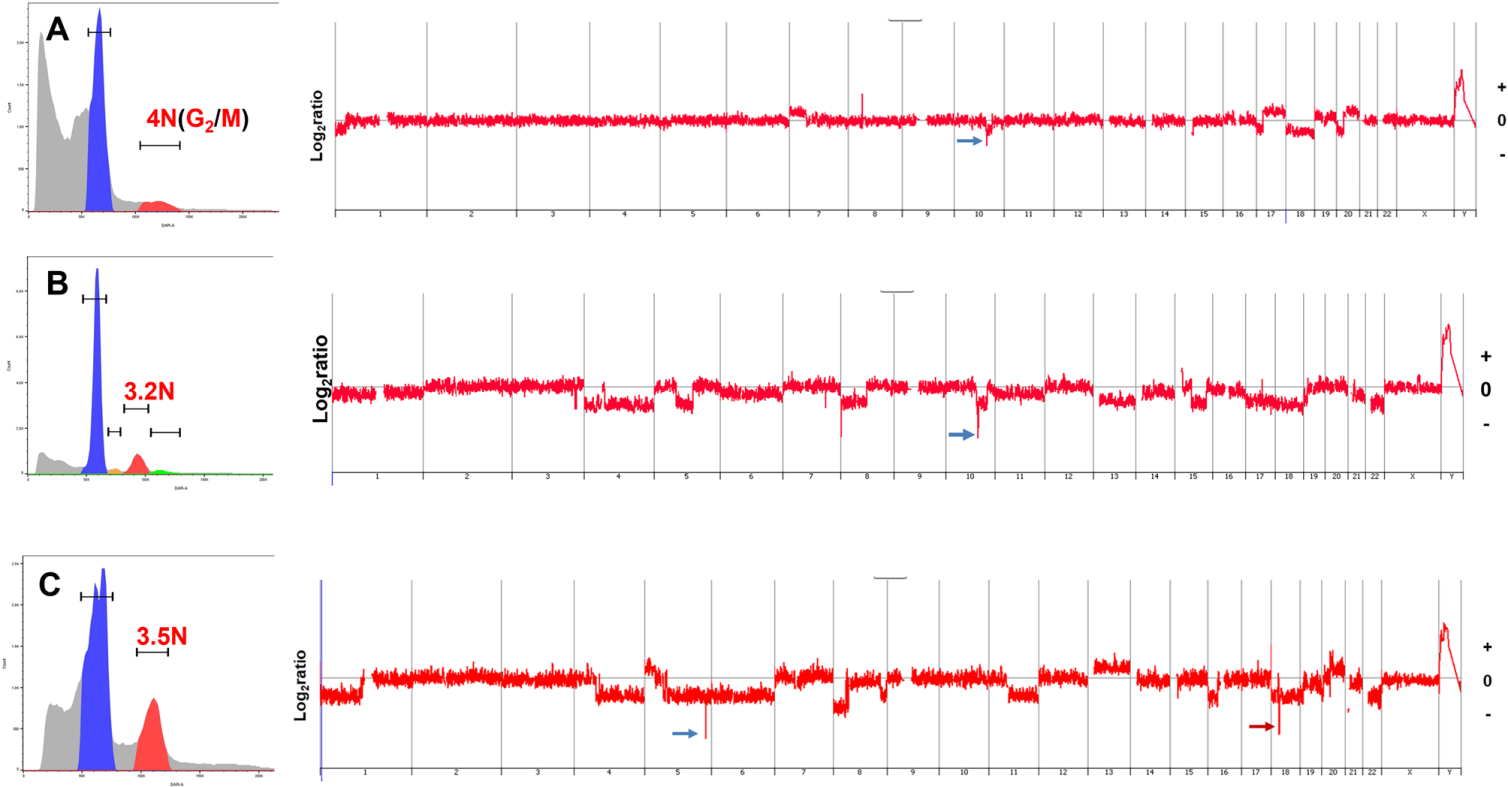
CNV profiles of EOCRC. (**A**) Diploid EOCRC3 with focal 10q23.31 *PTEN* deletion (blue arrow). (**B**) Aneuploid (3.2N) population sorted from EOCRC12 with focal 10q23.31 *PTEN* deletion (blue arrow). (**C**) Aneuploid (3.5N) population sorted from EOCRC17 with focal 5q34 deletion (blue arrow) and focal 18q11 deletion (red arrow).

The copy number variant (CNV) profiles in the six cases with sufficient tumor available after sorting and sequencing included a diploid tumor and five aneuploid cases (Fig. 5). For the diploid tumor we used the 4N(G_2_/M) fraction from the sort for CNV analysis, while the aneuploid tumors had distinct DNA contents. Notably, two cases, diploid (EOCRC3) and aneuploid (EOCRC12), had a focal deletion of *PTEN*. In addition, aneuploid case EOCRC17, a 37 year old female, had focal HDs that included known and novel cancer related genes (Supplemental Fig. 2). These included a 5q34 HD targeting *TENM2*/*ODZ2* (Teneurin Transmembrane Protein 2) that enables cell adhesion molecule binding activity and signaling receptor binding activity, and a 18q11 HD that simultaneously targets five genes with distinct functions; *PSMA8* (Proteasome 20S Subunit Alpha 8, *TAF4B* (TATA-Box Binding Protein Associated Factor 4b), *SS18* (SS18 Subunit Of BAF Chromatin Remodeling Complex), *ZNF521* Transcription factor, and *KCTD1* (potassium channel tetramerization domain containing 1). To our knowledge these two HDs have not been previously reported in primary tumor samples^34^.

### Telomere lengths

In each available case we compared the telomere lengths of diploid (P2) with either 4N(G_2_/M) or aneuploid(s) tumor populations (P3, P4, or P5). We detected a significant shortening of telomere lengths in the tumor fractions of the 9 samples assayed (two tailed paired sample t-test *P* = 0.00132) (Supplemental Fig. 3). Our application of single tube MMqPCR with patient matched flow sorted fractions controls for sample and assay variabilities as previously described^26^. Notably, the coefficient of variation (CV) within triplicate samples for each flow sorted population was less than 9.34%.

## Discussion

The prevalence of diploid (48%) and aneuploid (52%) tumors in our cohort is consistent with prior FISH studies supporting the enriched presence of diploid tumors in MSS EOCRC^17^. Our findings are also consistent with TCGA studies of later onset CRC that identified the WNT, MAPK, PI3K, TGF-band and p53 pathways as targets of somatic genomic lesions^10^. These included recurrent pathogenic mutations in *APC*, *TP53*, and *KRAS*, as well as deletions targeting *PTEN* that are present in our EOCRC samples. Targeted panel-based sequencing of large cohorts demonstrated similar mutational rates and TMBs in EOCRC and late onset cases with the most frequent alterations targeting *APC*, *TP53*, and *KRAS*^35^. In addition, multi-omic studies that include exome and genome analyses of different cohorts showed similar mutational profiles between early and late onset CRC but with enrichment of *PTEN* mutations in EOCRC^36^. The presence of focal *PTEN* deletions in two of six samples profiled for CNVs is notable given the association of *PTEN* mutations with EOCRC^36^. However, larger studies incorporating our flow cytometry methods are needed to determine whether *PTEN* lesions, both deletions and mutations, are enriched in EOCRC relative to average onset CRC.

CRC is distinguished by 4 distinct single base pair (SBS) mutational signatures^37^. We confirmed 3 of these signatures, SBS1, SBS5, and SBS6, but an absence of SBS10 in our EOCRC cohort. SBS10, defined by the presence of huge numbers of mutations in subsets of colorectal and uterine cancer, has been associated with altered activity of the error-prone polymerase Pol ε consequent on mutations in the gene^32^. The two POLE variant cases, EOCRC5 and EOCRC7 both somatic in nature, had the highest TMBs including the highest number of POLE associated mutations in our cohort (Supplemental Table 1). However, the total number of these variants did not meet criteria for scoring SBS10. Furthermore, the allele fraction for each variant (R793H, L1245I) was only 0.32 in both cases, one diploid (EOCRC5) and one aneuploid (EOCRC7) tumor, suggesting a sub clonal population in each case. Future single cell level studies with flow sorted clinical samples and preclinical models will provide additional insights related to the role of POLE variants in EOCRC.

Our TMB analyses discriminated 9 cases (43%) with relatively high mutation burden and 12 cases (57%) with low mutation burden (Fig. 2B). These were further distinguished by the presence of SBS5 in the high TMB cases and its absence in the low TMB cases (Fig. 4B,C). Notably, neither *TP53* mutation nor aneuploidy was associated with TMB. The presence of multiple aneuploid populations has been associated with accelerated progression in both premalignant and invasive carcinomas^38,39^. Although limited to single biopsies per case, we detected two co-occurring ploidies in EOCRC12 (Fig. 1B). Strikingly, although sharing the same mutation signatures, and *KRAS*^G12D^ and *APC*^D1486Yfs*27^ driver mutations, the 2.5N population had a > threefold increase in TMB relative to the 3.2N population (Fig. 2B). Future studies will include additional biopsies from individual cases to explore of multiple aneuploid populations and accelerated mutational processes in the evolution of EOCRC.

Our CNV analysis, although limited, highlights additional distinguishing features of EOCRC. These include *PTEN* deletions in two cases (Fig. 5). In addition, EOCRC17 a stage III sigmoid tumor from a 37 year old woman, had two unique HDs at 5q34 and 18q11 in the aneuploid tumor genome. The 5q34 deletion targets *TENM2* a member of the Teneurin gene family of transmembrane proteins that mediate cell–cell and cell-extracellular matrix interactions associated with important functions in development and nervous system function^40,41^. Gene-based expression-profile analyses from the Human Protein Atlas data sets suggest that *TENM2* expression has potential relevance as a prognostic marker in a range of tumors. Notably, low levels of *TENM2* expression are correlated with lower patients’ overall survival for colorectal, pancreatic, prostate and ovarian cancers. However, to our knowledge genomic lesions in *TENM2* have not been reported in EOCRC.

The five genes targeted by the 18q11 HD include the transcription factor *ZNF521* that has been previously reported in CRC based on single nucleotide polymorphism array profiling of patient derived xenografts that were used to overcome stromal tissue in patient samples^42^. In contrast, our flow-sorting samples provides discrimination of HDs directly in patient samples regardless of tumor/normal cell content in each biopsy of interest. This increased resolution mapped the four additional genes, *PSMA8*, *TAF4B*, *SS18*, and *KCTD1* within the same 18q11 HD (Supplemental Fig. 2). *ZNF521* regulates expression of RNA polymerase II, is involved in BMP signaling, and in the regulation of the immature compartment of the hematopoietic system and can both act as an activator or a repressor depending on the context^43^. It associates with SMADs in response to BMP2 leading to activate transcription of BMP target genes. *PSMA8* is a testis specific proteosome that promotes acetylation dependent degradation of histones and the degradation of meiotic proteins RAD51 and RPA1^44^. High protein levels are associated with good prognosis in breast cancer^45^. *TAF4B* is a component of a highly conserved regulatory network that promotes oocyte development^46^. This includes the proper development and morphogenesis of the embryonic intestinal endoderm^47^. Loss of *TAF4* in a mouse model led to increased PRC2 activity in cells of adult crypts associated with modification of the immune/inflammatory microenvironment that potentiated *APC*-driven tumorigenesis. Notably, EOCRC17 has an APC somatic R283X pathogenic nonsense variant. Genomic lesions targeting *SS18*, synovial sarcoma translocation chromosome 18, are associated with a variety of soft tissue tumors as well as a subset of CRC^48^. The most frequent events targeting *SS18* are fusions of amplifications. However, deletion of *SS18* has also been reported in a small subset (0.14%) of cancers. The fifth gene in the HD interval, *KCTD1*, negatively regulates the AP-2 family of transcription factors and the WNT signaling pathway^49,50^. Thus, this single HD deletes a series of genes and targets pathways with potential roles in EOCRC that will be explored in future studies.

Our current study confirms that EOCRCs present with different combinations of genomic lesions including aneuploidy, CNVs and variable TMB in the presence of driver mutations associated with average onset CRC related genes and pathways. These include a tumor (EOCRC12) with two distinct aneuploid populations that shared clonal driver mutations but distinct TMBs, a diploid tumor (EOCRC5) with relatively high TMB, and an aneuploid tumor (EOCRC7) with a relatively high TMB but a lack of CNVs (Supplemental Fig. 4). Furthermore, the presence of clock-like and age related mutation signatures SBS1 and SBS5 in the absence of MSI+, and the relative shortening of telomeres in tumor populations within a biopsy, add support to the model whereby EOCRCs result from aberrant accelerated biological aging^51^. However, despite the heterogenous nature of EOCRC, our IHC results and the presence of *LZTR1* variants and *PTEN* focal deletions, suggest that therapies targeting EGFR and AKT/mTOR pathway signalling may be of clinical benefit for distinct subsets of patients with this disease.

Our flow sorting approach provides a framework for detailed analyses of EOCRC. This includes discriminating and comparing diploid and aneuploid tumors with variable mutational and CNV burdens from archived tissue banks and/or prospectively collected biopsies. A challenge in the use of archived tumor samples is the presence of sequence artefacts and variable DNA quality in FFPE tissue. However, our established flow sorting protocols to isolate intact nuclei, GATK package tools for filtering FFPE artefacts from NGS data, and matching diploid and aneuploid fractions from single biopsies, provide a rigorous control and pipeline for genomic and telomere length analyses in our study^21,22,52,53^. The presence of unique HDs in the small number of samples profiled for CNVs suggests that more comprehensive studies incorporating whole genome sequencing of flow sorted samples will reveal additional examples of clinically relevant pathways targeted by genomic lesions in EOCRC. Previous studies have suggested that DNA methylation signatures contribute to EOCRC^54^. Of significant interest in our ongoing studies will be to integrate epigenetic profiling in our EOCRC analyses with flow sorted samples similar to our studies of pancreatic cancer^22^. Notably, the role of environmental exposures including microbiomes as well as predisposition genes and pathways will be explored in both premalignant and malignant models. These will provide novel mechanistic studies of the genes and pathways targeted in EOCRC, and promote the development of effective interventions to prevent and treat EOCRC.

### Supplementary Information

Supplementary Information.

## Data Availability

All exome sequencing data is available from (SRA accession number SUB13728991) https://www.ncbi.nlm.nih.gov/sra/PRJNA1019827. All CNV data have been deposited to GEO repository (GSE240339). All other data are available upon request from corresponding author.

## References

[1] Siegel RL, Wagle NS, Cercek A, Smith RA, Jemal A (2023). Colorectal cancer statistics, 2023. CA A Cancer J. Clin..

[2] Sinicrope FA (2022). Increasing Incidence of early-onset colorectal cancer. N. Engl. J. Med..

[3] Done JZ, Fang SH (2021). Young-onset colorectal cancer: A review. World J. Gastrointest. Oncol..

[4] Baran B, Mert Ozupek N, Yerli Tetik N, Acar E, Bekcioglu O, Baskin Y (2018). Difference between left-sided and right-sided colorectal cancer: A focused review of literature. Gastroenterol. Res..

[5] Dharwadkar P, Zaki TA, Murphy CC (2022). Colorectal cancer in younger adults. Hematol. Oncol. Clin. N. Am..

[6] Ahnen DJ, Wade SW, Jones WF, Sifri R, Mendoza Silveiras J, Greenamyer J (2014). The increasing incidence of young-onset colorectal cancer: a call to action. Mayo Clin. Proc..

[7] Bleyer A, Barr R, Hayes-Lattin B, Thomas D, Ellis C, Anderson B (2008). The distinctive biology of cancer in adolescents and young adults. Nat. Rev. Cancer.

[8] Chang DT, Pai RK, Rybicki LA, Dimaio MA, Limaye M, Jayachandran P (2012). Clinicopathologic and molecular features of sporadic early-onset colorectal adenocarcinoma: An adenocarcinoma with frequent signet ring cell differentiation, rectal and sigmoid involvement, and adverse morphologic features. Mod. Pathol..

[9] Yaeger R, Chatila WK, Lipsyc MD, Hechtman JF, Cercek A, Sanchez-Vega F (2018). Clinical sequencing defines the genomic landscape of metastatic colorectal cancer. Cancer Cell.

[10] The Cancer Genome Atlas Network (2012). Comprehensive molecular characterization of human colon and rectal cancer. Nature.

[11] Haan JC, Labots M, Rausch C, Koopman M, Tol J, Mekenkamp LJ (2014). Genomic landscape of metastatic colorectal cancer. Nat. Commun..

[12] Wood LD, Parsons DW, Jones S, Lin J, Sjoblom T, Leary RJ (2007). The genomic landscapes of human breast and colorectal cancers. Science.

[13] Linnekamp JF, Wang X, Medema JP, Vermeulen L (2015). Colorectal cancer heterogeneity and targeted therapy: A case for molecular disease subtypes. Cancer Res..

[14] Piawah S, Venook AP (2019). Targeted therapy for colorectal cancer metastases: A review of current methods of molecularly targeted therapy and the use of tumor biomarkers in the treatment of metastatic colorectal cancer. Cancer.

[15] Kirzin S, Marisa L, Guimbaud R, De Reynies A, Legrain M, Laurent-Puig P (2014). Sporadic early-onset colorectal cancer is a specific sub-type of cancer: A morphological, molecular and genetics study. PLoS One.

[16] Tricoli JV (2020). Genomic and molecular alterations associated with early-onset and adolescent and young adult colorectal cancer. Colorectal Cancer.

[17] Boardman LA, Johnson RA, Petersen GM, Oberg AL, Kabat BF, Slusser JP (2007). Higher frequency of diploidy in young-onset microsatellite-stable colorectal cancer. Clin. Cancer Res..

[18] Kneuertz PJ, Chang GJ, Hu CY, Rodriguez-Bigas MA, Eng C, Vilar E (2015). Overtreatment of young adults with colon cancer: More intense treatments with unmatched survival gains. JAMA Surg..

[19] You YN, Lee LD, Deschner BW, Shibata D (2020). Colorectal cancer in the adolescent and young adult population. JCO Oncol. Pract..

[20] Cercek A, Chatila WK, Yaeger R, Walch H, Fernandes GDS, Krishnan A (2021). A comprehensive comparison of early-onset and average-onset colorectal cancers. J. Natl. Cancer Inst..

[21] Holley T, Lenkiewicz E, Evers L, Tembe W, Ruiz C, Gsponer JR (2012). Deep clonal profiling of formalin fixed paraffin embedded clinical samples. PLoS One.

[22] Lenkiewicz E, Malasi S, Hogenson TL, Flores LF, Barham W, Phillips WJ (2020). Genomic and epigenomic landscaping defines new therapeutic targets for adenosquamous carcinoma of the pancreas. Cancer Res..

[23] Ruiz C, Lenkiewicz E, Evers L, Holley T, Robeson A, Kiefer J (2011). Advancing a clinically relevant perspective of the clonal nature of cancer. Proc. Natl. Acad. Sci. USA.

[24] Barrett MT, Deiotte R, Lenkiewicz E, Malasi S, Holley T, Evers L (2017). Clinical study of genomic drivers in pancreatic ductal adenocarcinoma. Br. J. Cancer.

[25] Lipson D, Aumann Y, Ben-Dor A, Linial N, Yakhini Z (2006). Efficient calculation of interval scores for DNA copy number data analysis. J. Comput. Biol..

[26] Cawthon RM (2009). Telomere length measurement by a novel monochrome multiplex quantitative PCR method. Nucleic Acids Res..

[27] Castel P (2022). Defective protein degradation in genetic disorders. Biochim. Biophys. Acta Mol. Basis Dis..

[28] Frattini V, Trifonov V, Chan JM, Castano A, Lia M, Abate F (2013). The integrated landscape of driver genomic alterations in glioblastoma. Nat. Genet..

[29] Ko A, Hasanain M, Oh YT, D'Angelo F, Sommer D, Frangaj B (2023). LZTR1 mutation mediates oncogenesis through stabilization of EGFR and AXL. Cancer Discov..

[30] Pabla B, Bissonnette M, Konda VJ (2015). Colon cancer and the epidermal growth factor receptor: Current treatment paradigms, the importance of diet, and the role of chemoprevention. World J. Clin. Oncol..

[31] Yu J, Zheng Y, Dong J, Klusza S, Deng WM, Pan D (2010). Kibra functions as a tumor suppressor protein that regulates Hippo signaling in conjunction with Merlin and expanded. Dev. cell.

[32] Alexandrov LB, Kim J, Haradhvala NJ, Huang MN, Tian Ng AW, Wu Y (2020). The repertoire of mutational signatures in human cancer. Nature.

[33] Hodel KP, Sun MJS, Ungerleider N, Park VS, Williams LG, Bauer DL (2020). POLE mutation spectra are shaped by the mutant allele identity, its abundance, and mismatch repair status. Mol. Cell.

[34] Cheng J, Demeulemeester J, Wedge DC, Vollan HKM, Pitt JJ, Russnes HG (2017). Pan-cancer analysis of homozygous deletions in primary tumours uncovers rare tumour suppressors. Nat. Commun..

[35] Lieu CH, Golemis EA, Serebriiskii IG, Newberg J, Hemmerich A, Connelly C (2019). Comprehensive genomic landscapes in early and later onset colorectal cancer. Clin. Cancer Res..

[36] Lee W, Wang Z, Saffern M, Jun T, Huang KL (2021). Genomic and molecular features distinguish young adult cancer from later-onset cancer. Cell Rep..

[37] Alexandrov LB, Nik-Zainal S, Wedge DC, Aparicio SA, Behjati S, Biankin AV (2013). Signatures of mutational processes in human cancer. Nature.

[38] Ben-David U, Amon A (2020). Context is everything: Aneuploidy in cancer. Nat. Rev. Genet..

[39] Martinez P, Mallo D, Paulson TG, Li X, Sanchez CA, Reid BJ (2018). Evolution of Barrett's esophagus through space and time at single-crypt and whole-biopsy levels. Nat. Commun..

[40] Tucker RP, Chiquet-Ehrismann R (2006). Teneurins: A conserved family of transmembrane proteins involved in intercellular signaling during development. Dev. Biol..

[41] Peppino G, Ruiu R, Arigoni M, Riccardo F, Iacoviello A, Barutello G (2021). Teneurins: Role in cancer and potential role as diagnostic biomarkers and targets for therapy. Int. J. Mol. Sci..

[42] Leary RJ, Lin JC, Cummins J, Boca S, Wood LD, Parsons DW (2008). Integrated analysis of homozygous deletions, focal amplifications, and sequence alterations in breast and colorectal cancers. Proc. Natl. Acad. Sci. USA.

[43] Bond HM, Mesuraca M, Carbone E, Bonelli P, Agosti V, Amodio N (2004). Early hematopoietic zinc finger protein (EHZF), the human homolog to mouse Evi3, is highly expressed in primitive human hematopoietic cells. Blood.

[44] Gomez HL, Felipe-Medina N, Condezo YB, Garcia-Valiente R, Ramos I, Suja JA (2019). The PSMA8 subunit of the spermatoproteasome is essential for proper meiotic exit and mouse fertility. PLoS Genet..

[45] Chiao CC, Liu YH, Phan NN, An Ton NT, Ta HDK, Anuraga G (2021). Prognostic and genomic analysis of proteasome 20S subunit alpha (PSMA) family members in breast cancer. Diagnostics (Basel).

[46] Grive KJ, Gustafson EA, Seymour KA, Baddoo M, Schorl C, Golnoski K (2016). TAF4b regulates oocyte-specific genes essential for meiosis. PLoS Genet.

[47] Saisa-Borreill S, Davidson G, Kleiber T, Thevenot A, Martin E, Mondot S (2023). General transcription factor TAF4 antagonizes epigenetic silencing by polycomb to maintain intestine stem cell functions. Cell Death Differ..

[48] Consortium APG (2017). AACR project GENIE: Powering precision medicine through an international consortium. Cancer Discov..

[49] Hu L, Chen L, Yang L, Ye Z, Huang W, Li X (2020). KCTD1 mutants in scalp-ear-nipple syndrome and AP-2alpha P59A in Char syndrome reciprocally abrogate their interactions, but can regulate Wnt/beta-catenin signaling. Mol. Med. Rep..

[50] Ding X, Luo C, Zhou J, Zhong Y, Hu X, Zhou F (2009). The interaction of KCTD1 with transcription factor AP-2alpha inhibits its transactivation. J. Cell Biochem..

[51] Boardman LA, Litzelman K, Seo S, Johnson RA, Vanderboom RJ, Kimmel GW (2014). The association of telomere length with colorectal cancer differs by the age of cancer onset. Clin. Transl. Gastroenterol..

[52] Phung TN, Lenkiewicz E, Malasi S, Sharma A, Anderson KS, Wilson MA (2020). Unique genomic and neoepitope landscapes across tumors: A study across time, tissues, and space within a single lynch syndrome patient. Sci. Rep..

[53] Phung TN, Webster TH, Lenkiewicz E, Malasi S, Andreozzi M, McCullough AE (2021). Unique evolutionary trajectories of breast cancers with distinct genomic and spatial heterogeneity. Sci. Rep..

[54] Joo JE, Clendenning M, Wong EM, Rosty C, Mahmood K, Georgeson P (2021). DNA methylation signatures and the contribution of age-associated methylomic drift to carcinogenesis in early-onset colorectal cancer. Cancers (Basel).

