# Supplementary material for: Genomic landscape of diploid and aneuploid microsatellite stable early onset colorectal cancer
Source: Sci Rep. 2024 Apr 23;14:9368. doi: 10.1038/s41598-024-59398-5 (PMC11039710; doi:10.1038/s41598-024-59398-5)
Supplement: Supplementary file 1 — Supplementary Information. [file 41598_2024_59398_MOESM1_ESM.pdf]

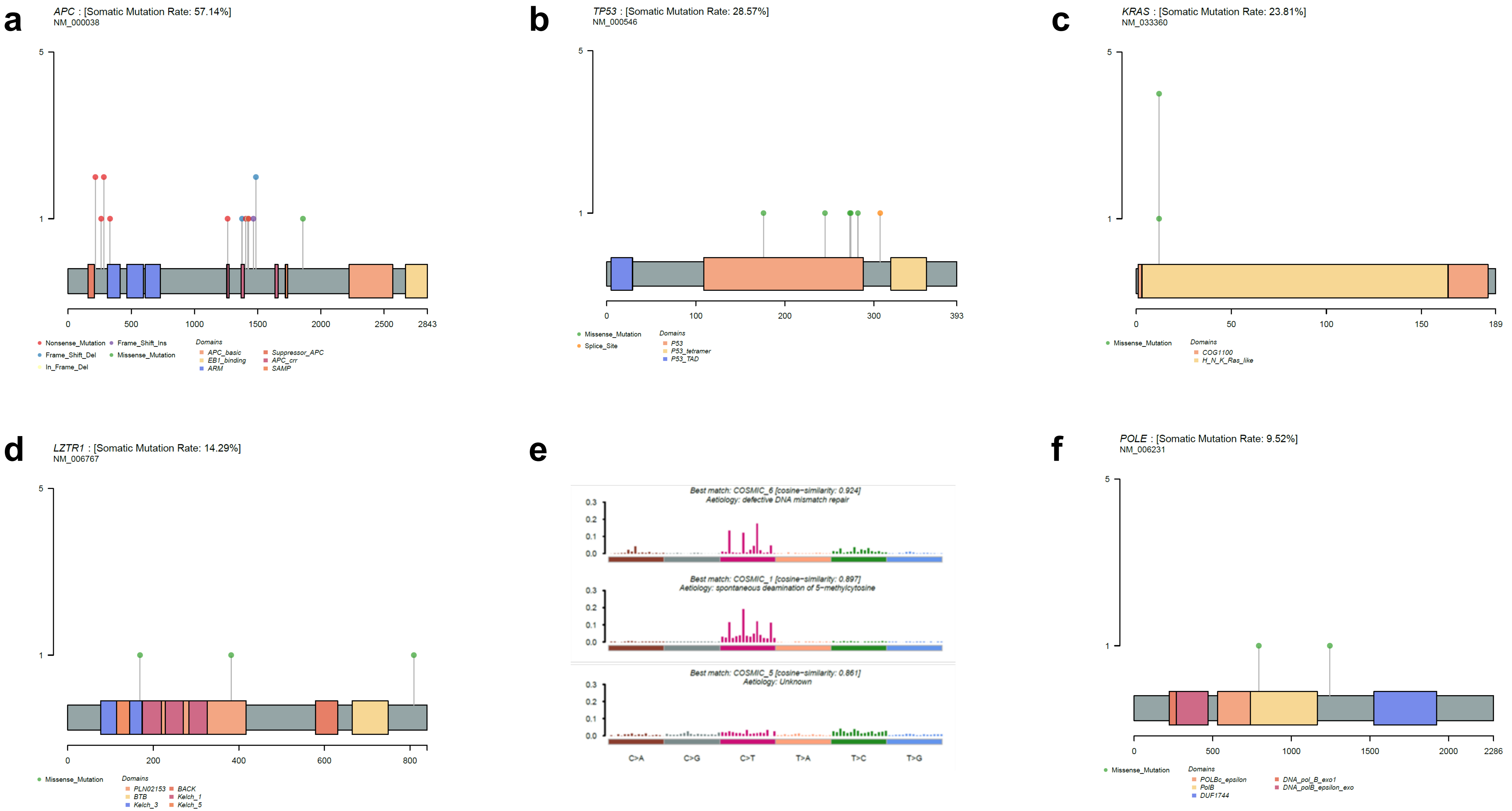

Supplemental Figure 1. Mutation profiles of EOCRC and related genes. **A**) APC, **B**) TP53, **C**) KRAS, **D**) LZTR1, **E**) SBS 1, 5, and 6, and **F**) POLE.

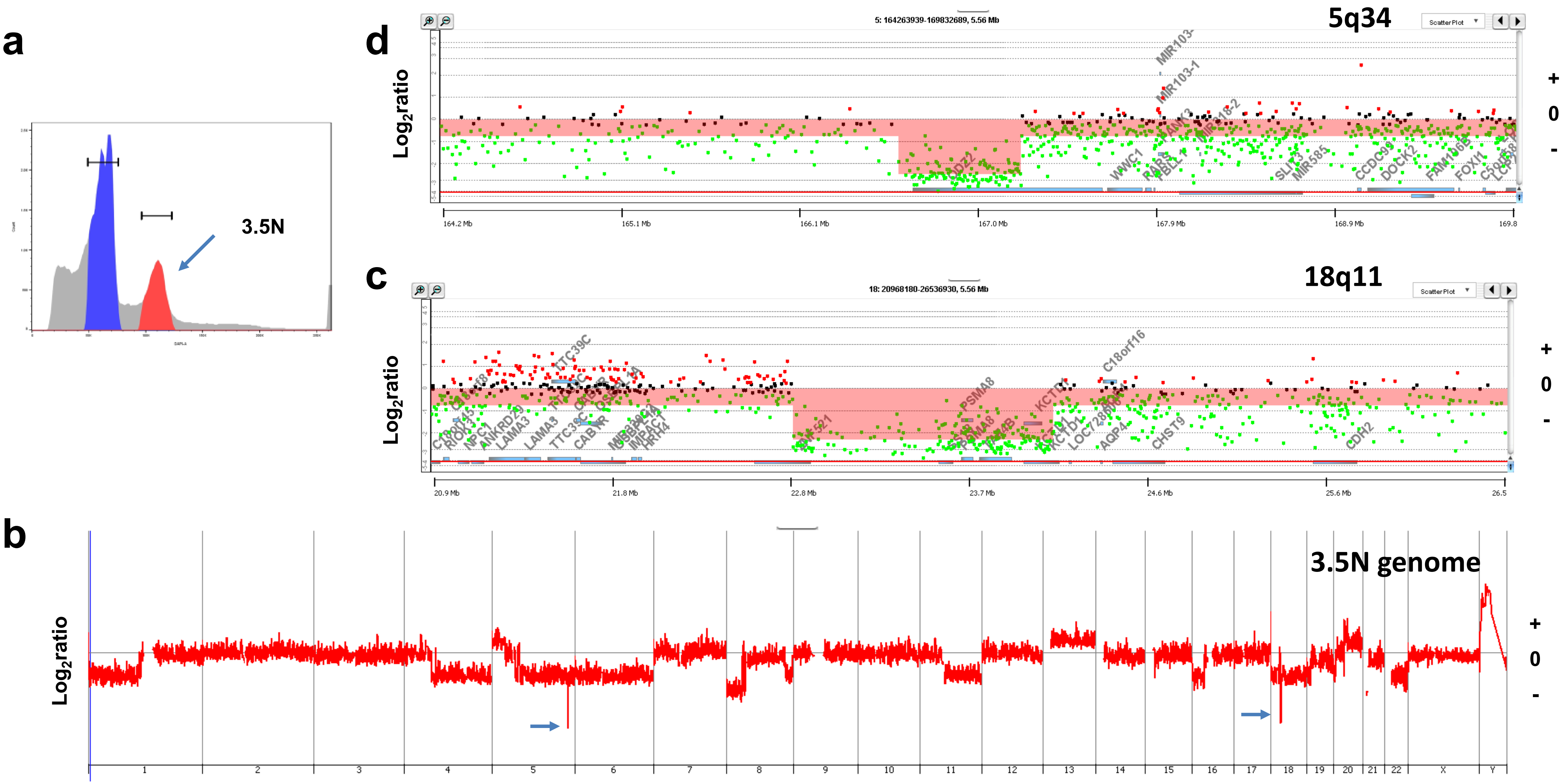

Supplemental Figure 2. Mapping of homozygous deletions in EOCRC17**. A)** DNA content flow-sorting histogram. **B)** Whole-genome CNV profile of the flow-sorted 3.5N population. Homozygous deletions at **C)** 18q11 targeting *ZNF521*, *SS18*, *PSMA8*, *TAF4B,* and *KCTD1* and **D)** 5q34 targeting *ODZ2*. The red shaded areas denote ADM2 step gram-defined regions of deletions.

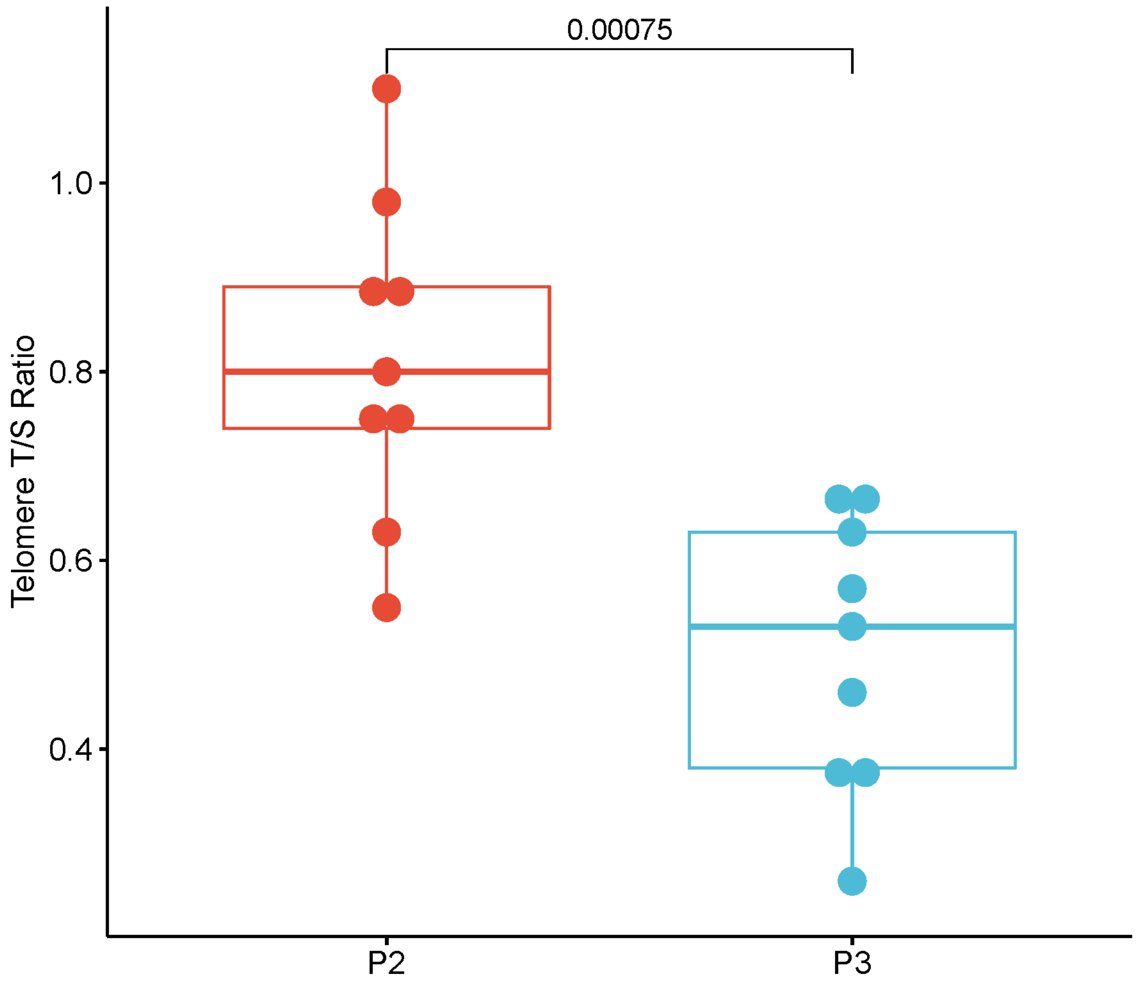

Sample ID	Telomere T/S Ratio
EOCRC03-P2	0.98
EOCRC07-P2	0.55
EOCRC11-P2	1.10
EOCRC12-P2	0.80
EOCRC13-P2	0.76
EOCRC14-P2	0.74
EOCRC15-P2	0.88
EOCRC17-P2*	0.89
EOCRC19-P2	0.63
EOCRC03-P3	0.67
EOCRC07-P3*	0.66
EOCRC11-P3	0.57
EOCRC12-P5	0.46
EOCRC13-P4	0.53
EOCRC14-P4	0.26
EOCRC15-P4	0.37
EOCRC17-P3*	0.63
EOCRC19-P4	0.38
*Ave. value from two measurements	

Supplemental Figure 3. Telomere length measurements of flow-sorted EOCRC tumors**.** The telomere single-copy gene (T/S) ratio of matching diploid (P2) and tumor (P3-P5) fractions from 9 EOCRC samples.

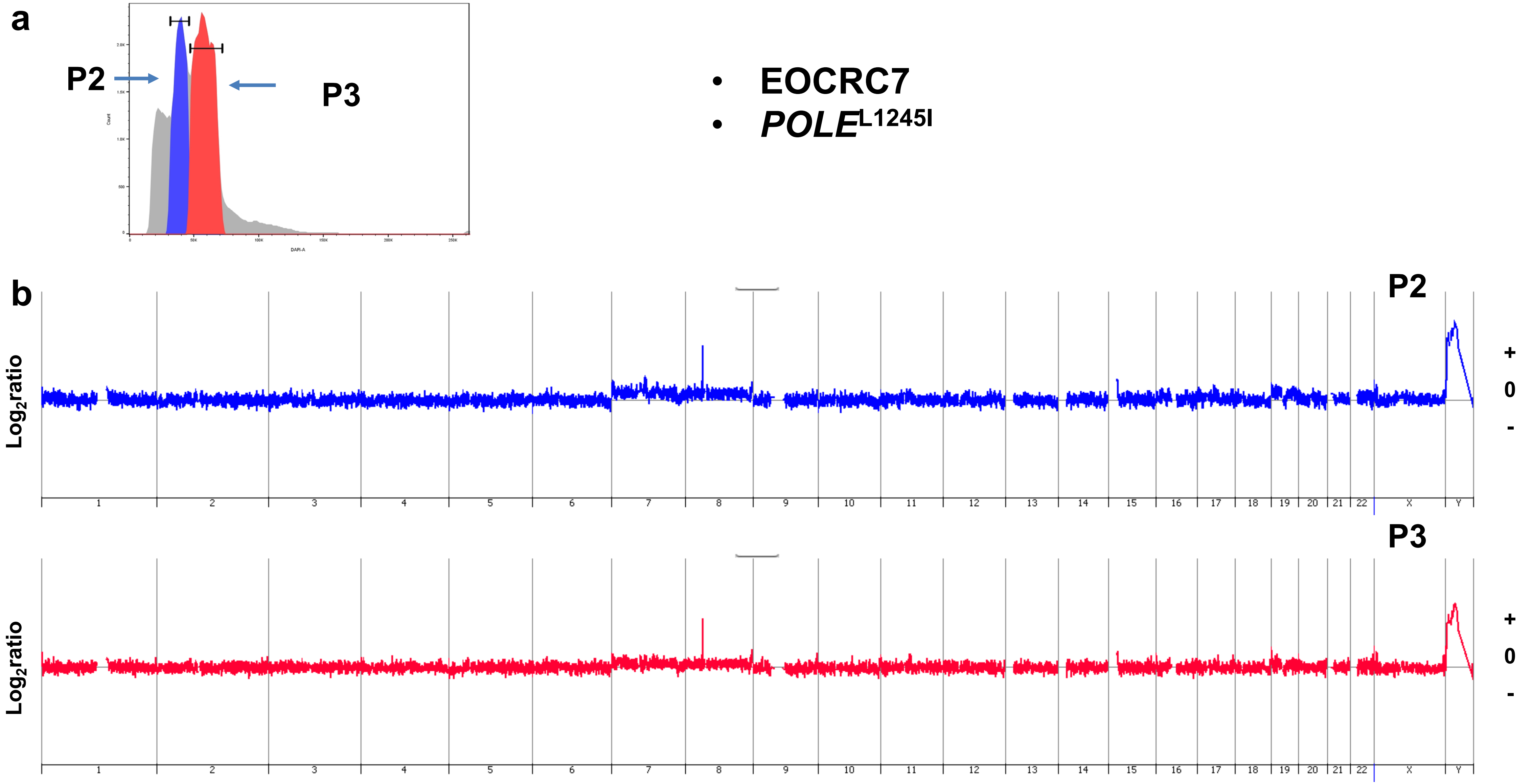

Supplemental Figure 4. CNV profiles of flow-sorted EOCRC7. A) Two populations (P2 and P3) were identified and sorted from FFPE tissue. B) The CNV profile for each sorted population was copy number neutral.

Supplemental Table 1: POLE Signature Mutations

Sample ID	B TCT.AGT	C TCT.A	D TCG.AGT	E TCG.T	F TTT.AGC	G TTT.G	B+D+F signature	C+E+G signature	Total Tumor mutations	% POLE signature mutations
EOCRC21	0	0	0	0	0	0	0	0	20	0.00
EOCRC15	0	0	0	0	0	0	0	0	4	0.00
EOCRC17	0	0	0	0	0	0	0	0	70	0.00
EOCRC01	0	0	1	1	0	0	1	1	42	2.38
EOCRC20	0	0	1	1	0	0	1	1	9	11.11
EOCRC12.AN2+	1	0	2	2	0	0	3	2	103	1.94
EOCRC16	0	0	2	2	0	0	2	2	58	3.45
EOCRC04	0	0	3	3	0	0	3	3	76	3.95
EOCRC14	0	0	5	5	1	0	6	5	86	5.81
EOCRC06	0	0	7	7	1	0	8	7	69	10.14
EOCRC18	3	2	6	6	0	0	9	8	300	2.67
EOCRC11	3	1	9	9	0	0	12	10	103	9.71
EOCRC03	3	1	10	9	0	0	13	10	107	9.35
EOCRC12.AN1+	6	0	7	7	5	4	18	11	382	2.88
EOCRC09	5	2	15	15	0	0	20	17	533	3.19
EOCRC13	10	10	9	8	4	0	23	18	156	11.54
EOCRC19	0	0	17	17	2	1	19	18	914	1.97
EOCRC10	14	3	21	18	3	1	38	22	763	2.88
EOCRC02	2	0	22	22	0	0	24	22	325	6.77
EOCRC08	2	0	29	29	0	0	31	29	440	6.59
EOCRC07*	5	2	36	36	0	0	41	38	1082	3.51
EOCRC05#	10	1	62	62	1	0	73	63	1004	6.27

+Co-occurring aneuploid populations
*$\textit{POLE}^{L1245I}$
#$\textit{POLE}^{R793H}$

Supplemental Table 2. EOCRC Cohort

Sample#	Sex	Age at Dx	Pathology	Stage	Site	Ploidy	EGFR Staining
EOCRC1	Female	43	ADCA	3	Cecum	2N/4N	1+
EOCRC2	Male	49	ADCA	3	Descending	2N/3N	2+
EOCRC3	Female	46	ADCA	2	Cecum	2N/4N	1+
EOCRC4	Male	39	ADCA	3	Rectosigmoid	2N/3.4N	0
EOCRC5	Male	43	ADCA	3	Rectum	2N/4N	0
EOCRC6	Male	44	ADCA	3	Rectosigmoid	2N/3.2N	0
EOCRC7	Female	40	ADCA	2	Cecum	2N/2.9N	3+
EOCRC8	Male	48	ADCA	2	Rectosigmoid	2N/3.5N	1+
EOCRC9	Female	41	ADCA	2	Rectum	2N/4N	3+
EOCRC10	Female	44	ADCA	2	Transverse	2N/4N	2+
EOCRC11	Male	31	ADCA	3	Rectum	2N/4N	0
EOCRC12	Female	49	ADCA	3	Ileocecal Valve	2N/2.5N[a]/3.2N[b]/4N	3+
EOCRC13	Male	38	ADCA	2	Rectum	2N/2.6N	1+
EOCRC14	Female	42	ADCA	3	Rectosigmoid	2N/2.6N	2+
EOCRC15	Male	42	ADCA	2	Cecum	2N/4N	3+
EOCRC16	Male	41	ADCA	3	Cecum	2N/4N	2+
EOCRC17	Female	37	ADCA	3	Sigmoid	2N/3.5N	0
EOCRC18	Male	45	ADCA	2	Ascending	2N/4N	1+
EOCRC19	Male	23	ADCA	2	Cecum	2N/2.4N	3+
EOCRC20	Male	32	ADCA	2	Hepatic Flexure	2N/4N	0
EOCRC21	Male	36	ADCA	2	Sigmoid	2N/3.2N	ND

EOCRC12: [a]AN1, [b]AN2
